# The emerging role of neutrophil extracellular traps in the progression of rheumatoid arthritis

**DOI:** 10.3389/fimmu.2024.1438272

**Published:** 2024-08-16

**Authors:** Jingjing Chen, Yang Cao, Jing Xiao, Yujie Hong, Yan Zhu

**Affiliations:** ^1^ The Geriatrics, Graduate School of Anhui University of Chinese Medicine, Hefei, China; ^2^ The Geriatrics, The Second Affiliated Hospital of Anhui University of Chinese Medicine, Hefei, China

**Keywords:** neutrophil extracellular trap, rheumatoid arthritis, autoantibody, citrullination, key role

## Abstract

Rheumatoid arthritis (RA) is a chronic autoimmune disease with a complex etiology. Neutrophil extracellular traps (NETs are NETwork protein structures activated by neutrophils to induce the cleavage and release of DNA-protein complexes). Current studies have shown the critical involvement of NETs in the progression of autoimmune diseases, Neutrophils mostly gather in the inflammatory sites of patients and participate in the pathogenesis of autoimmune diseases in various ways. NETs, as the activated state of neutrophils, have attracted much attention in immune diseases. Many molecules released in NETs are targeted autoantigens in autoimmune diseases, such as histones, citrulline peptides, and myeloperoxidase. All of these suggest that NETs have a direct causal relationship between the production of autoantigens and autoimmune diseases. For RA in particular, as a disorder of the innate and adaptive immune response, the pathogenesis of RA is inseparable from the generation of RA. In this article, we investigate the emerging role of NETs in the pathogenesis of RA and suggest that NETs may be an important target for the treatment of inflammatory autoimmune diseases.

## 1 Introduction

Rheumatoid arthritis (RA) is a systemic immune disorder mainly characterized by erosive joint damage involving the hands, wrists, ankles, knees, and other joints. In the early stage, the joints may become red, swollen, hot, and dysfunctional, and gradually deteriorate to stiffness and deformation in the late stage. At present, the pathogenesis of RA remains unclear, and its main pathological manifestations include immune cell infiltration, synovium hyperplasia and formation, and articular cartilage and bone destruction. Accumulating studies have been conducted regarding the molecular mechanisms of RA. There are various breakthrough points for RA molecular mechanism research, and, among its autoantibodies, anti-citrullinated protein antibody (ACPA) is an important factor that cannot be ignored in RA disease development (1). With the increase in global life expectancy, the number of patients with RA is also rising, and the proportion of female patients is approximately three times that of male patients (2, 3). The incidence of RA is 0.5%–1%, affecting 0.2–1% of the global population. A positive family history can increase the risk of RA by approximately 3–5 times, and genetic factors cannot be ignored as a cause of RA (3). Studies have found that compared with non-RA patients, RA patients have a higher probability of suffering from multiple diseases (approximately 31–86%), a faster accumulation of comorbidity, a worse prognosis, a higher risk of disease activity, and a higher possibility of biologic agent failure. These data indicate that the clinical treatment of RA is urgent (4). Currently, disease-modifying anti-rheumatic drugs are still the first choice treatment for RA, and non-steroidal anti-inflammatory drugs and glucocorticoids have also been proven to reduce RA inflammation and pain (5). However, these drugs are also accompanied by inevitable shortcomings, such as side effects, adverse reactions, and drug resistance, and cannot really cure RA. Almost all RA patients need lifelong treatment; therefore, there is an urgent need for new treatment methods to delay the pathological process of RA and provide new therapeutic targets. Existing studies have pointed out the critical implication of neutrophils in the development of RA, and neutrophils abundantly exist in the synovial fluid and synovial tissue of RA joints (6, 7), while neutrophil extracellular traps (NETs) are important sources of citrullinated proteins and cytokines in RA and contribute to promoting the formation of ACPA. Additionally, NETs can promote the proliferation of synovial fibroblasts and produce cytokines, thus aggravating joint inflammation. Therefore, this article will explore the pathogenesis and role of NETs in RA and consider the potential and challenge of NETs as a new treatment strategy for RA, with a view to opening up new ways to prevent and treat RA.

## 2 Overview of NETs

### 2.1 The formation of NETs

Neutrophils are stimulated by phobolate (PMA) and lipopolysaccharide (LPS). Brinkmann et al. found that the NETswork structure formed by chromatin in neutrophils had the potential of being an antibacterial barrier, and such NETswork structures containing DNA-protein were designated as NETs (8). NETs are fibrous structures released by neutrophils in response to specific stimuli. These structures are composed of neutrophil particles and decondensed chromatin coated with cytoplasmic proteins (9). NETs mainly bind neutrophil elastase (NE), myeloperoxidase (MPO), histone sphaerolysis concentrated chromatin, Caldwell protein, and defensin to capture and kill bacteria or viruses (8, 10). In the current situation regarding autoimmune diseases, a detailed proteomic analysis focusing on the composition of specific NETs holds promise for revealing new mechanisms underlying disease onset and progression (11).

### 2.2 The NETosis pathway of NETs release

NETosis is a peculiar death modality of neutrophils that gives rise to the production of NETs (12), which contribute to the neutralization of invading pathogens and restoration of homeostasis. There are multiple stimuli that trigger NETosis but the mechanism by which NETosis releases NETs is not exactly the same. In spite of this, chromatin deconcentration is a necessary condition for NETosis (13). Here, we describe three pathways by which NETosis releases NETs (**Table 1**).

**Table 1:** The difference between the three pathways of NETosis generation.

NETosis pathway category	Stimulating factor	Conductor receptor	Trigger key	DNA cleavage	Is the nuclear membrane intact
Suicidal NETosis	Virus、 fungus、 PMA	FcR	NADPH、 NOX2、 NE、 MPO	Nuclear DNA	No
Active NETosis	Virus (*S. aureus*)、 fungus	TLR2/TLR4	Mitochondrial ROS、 Ca+、 NE、 MPO	Nuclear DNA	Yes
Mitochondrial NETosis	CMS-CF/Ca5/LPS	TLR4	Mitochondrial ROS、 NE、 MPO	Mitochondrial DNA	NO

**Figure 1:**
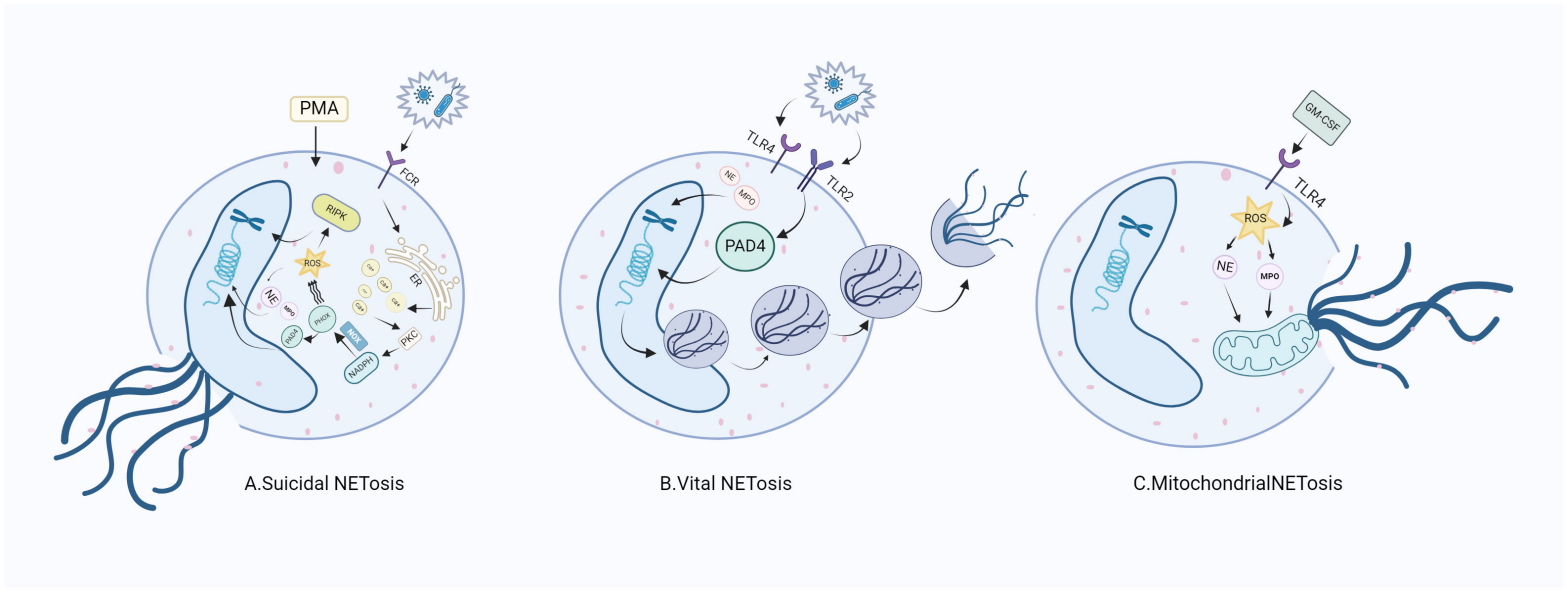
NETosis releases NETs through three different mechanisms. **(A)** After neutrophils are stimulated, ROS production is induced through the NADPH pathway to stimulate nuclear DNA cleavage, initiating the release of NE and MPO in neutrophils, which intensifies the direct cleavage of nuclear DNA and directly destroys the cell membrane, thus releasing NETs. **(B)** When stimulated, neutrophils do not depend on NADph-dependent ROS, but intracellular mitochondrial ROS can directly promote the activation of PAD4 and the release of NE and MPO, thus promoting the nuclear DNA wrapped in vesicles to release NETs. **(C)** Neutrophils generate NETs through the cleavage of mitochondrial DNA after stimulation. RA, rheumatoid arthritis; FCR, fragment crystallizable receptor; PMA, phorbo nutmeg acetate; ER, endoplasmic reticulum; PKC, protein kinase C; NADPH, nicotinamide adenine dinucleotide phosphate oxidase; NOX, nitric oxide; PIPK3: receptor interacting protein kinase 3; PAD4, peptidylarginine deiminase 4; NE, neutrophil elastase; MPO, myeloperoxidase; NETs, neutrophil extracellular traps; ROS, reactive oxygen species; TLR, toll-like receptor; GM-CSF, granulocyte macrophage colony-promoting factor.

Suicidal NETosis, also known as NADPH oxidase-dependent NETs, is triggered by neutrophil receptors [such as toll-like receptor (TLR)] recognizing a variety of stimuli (such as bacteria, viruses, and fungi) and persists for a long time. Upon receiving the stimulation, calcium ions are first released from the endoplasmic reticulum (ER) into the cytoplasm, which increases the activity of protein kinase C (PKC) and induces nicotinamide adenine dinucleotide phosphate (NADPH) to form a functional complex (PHOX) through the RAF-MERK-ERK signaling pathway (14), leading to the production of reactive oxygen species (ROS) and the activation of receptor interacting protein kinase 3 (RIPK3) and mixed lineage kinase domain-like protein (MLKL). NADPH oxidase (NOX) activates PAD4, an enzyme downstream of ROS and calcium signals (15, 16). NE and MPO are released from azinotropic particles and transferred into the nucleus with the assistance of NADPH. The cleavage protein promotes chromatin decondensation, so that the decondensed chromatin containing cytoplasmic and granular components is excreted out of the cell (17), thus initiating programmed cell death (18). Relevant clinical evidence proves that suicidal NETosis requires the involvement of ROS. Neutrophils lacking sufficient ROS isolated from patients with chronic granulomatous disease fail to produce NETs under stimulation (19), and the use of NOX inhibitors can prevent the formation of NETs (20, 21) (**Figure 1A**).

Active NETosis, also known as non-NADPH oxidation-dependent NETs, is characterized by the fact that the nuclear membrane of neutrophils remains intact and active after the release of NETs, and neutrophils continue to migrate to bacteria, a process crucial for resisting bacterial invasion (22). NETs under this mechanism are released rapidly after exposure to *Staphylococcus aureus* through the secretion of chromatin and particles (23). Under this pathway, the formation of NETs can occur independently of the production of NADPH oxidase ROS. Specifically, PAD4 is activated in the presence of elevated levels of mitochondrial ROS and intracellular calcium (24), resulting in histone citrullination and chromatin decondensation, in which the DNA-protein complex envelopes the vesicles and releases its load outside the cell, without breaking the nuclear membrane in the process. To our knowledge, the difference between suicidal NETosis and critical NETosis lies in the dependence of the two approaches to NOX and whether the nuclear membrane is intact after chromatin release (**Figure 1B**).

The third form of NETosis (mitochondrial NETosis) relies on the production of ROS, with living cells formed from mitochondrial DNA releasing NETs, instead of nuclear DNA. Mitochondrial DNA causes neutrophils to release NETs in response to granulocyte-macrophage colony-stimulating factor (GM-CSF) pre-treatment and LPS/C5a stimulation while retaining their nuclei (25, 26). Mitochondrial NETosis has also been observed in LPS-stimulated neutrophils isolated from trauma patients after orthopedic surgery (27). As reports on mitochondrial NETosis are currently limited, further studies on the mechanism of mitochondrial NETosis are needed to gain a deeper understanding (28). (**Figure 1C**).

### 2.3 Key factors triggering NETosis

Neutrophil activation is the first condition for NETosis production, and resting neutrophils under non-inflammatory conditions do not develop NETosis. Neutrophils can induce NETosis at surface pattern recognition receptors (29). In addition to the relevant receptors, NETosis can also be induced by bacterial toxins, such as ionomycin (30) and Nigericin (31). Therefore, neutrophil activation requires the involvement of bacterial toxins or surface receptors to initiate NETosis (32). A large number of activated neutrophils and high levels of neutrophil granulocytes have also been found in the synovial and joint tissues of RA patients, which aggravate the proliferation and invasion of synovial cells activating cytokines and receptors to participate in the pathogenesis of RA (33), and relevant experiments have also yielded compelling evidence to support the activation of neutrophils and formation of NETs during the development of RA (34).

Changes in calcium concentration are also key to NETosis activation. Neutrophil activation will lead to an increase in intracellular calcium concentration, and the binding of ligand and complement receptor will trigger the ER to release stored calcium, and then open the plasma membrane calcium channel, resulting in a disturbance in the calcium ion balance. The increase of intracellular Ca2+ has been demonstrated to be a prerequisite for the production of NETs induced by optimal PMA (31, 35). In the context of NETosis, an increase in intracellular calcium has been observed in neutrophils induced by LPS, IL-8, and PMA, as well as by the calcium ion carriers iomycin and A23187, leading to the formation of NETs (36). Therefore, the increase of intracellular calcium, whether the calcium released from intracellular storage or the calcium flowing into the cell from the extracellular environment, is critical for NETosis.

PAD4, an enzyme mediated by calcium, plays a crucial role in NETosis (37). It is generally believed that PAD4 drives NETosis through citrullinated histones. PAD4 is highly expressed in neutrophils and ectopic to the nucleus in the presence of elevated cytosolic calcium concentration, and mediates the conversion of arginine residues to citcitline in the target protein (38). The internal force of PAD4 on citrullinated histones and its specific expression make it an important link mediating chromatin decondensation during NETosis (32). PAD4 itself is the target of autoantibodies and shows a high-expression profile in the synovium of RA. A positive correlation between the polymorphism of *PAD4* gene and the incidence of RA has been revealed (39, 40), which is better explained by the fact that the PAD4 inhibitor chloropyrimidine can alleviate the symptoms of collagen-induced arthritis (CIA) in mice and rats (41).

ROS production is also an important step involved in the formation of NETosis. ROS in neutrophils are mainly derived from NOX and mitochondria. Stimulating neutrophils with PMA, *S. aureus*, or group B *Streptococcus* can generate ROS within 20 min, and neutrophils with ROS scavengers can inhibit PMA-induced NETosis (20, 31). Neutrophils lacking functional NOX cannot develop NETosis under the stimulation of *S. aureus* or PMA (19). However, the exact mechanism by which NOX drives the release of NETs remains unclear (32). It has been shown that calcium ion-induced NOX-dependent NETosis is mediated by mitochondrial ROS (24). ROS are indispensable in all forms of NETosis and can promote the release of NE and MPO, thus accelerating the process of NETosis. However, its specific mechanism still needs further exploration.

## 3 The relationship between NETs and RA

Plenty of autoantibodies have been identified as markers of RA, including rheumatoid factor (RF) and antibodies against post-translational modified proteins [such as anti-citrullinated protein autoantibody (ACPA)] and carbamylation, which may form immune complexes within the joint and lead to the aggregation of immune cells (42). Compared with healthy individuals, RA patients present detectable activated neutrophils in the circulation, which can last for several days and accumulate in large numbers in the synovial fluid and vascular membranes of RA, leading to RA inflammation and joint destruction. The increase of neutrophils is associated with increased ROS production, elevated MPO expression, and increased citrullination mediated by PAD4 activity. Therefore, ROS, the NOX pathway, and PAD4 activity are important factors in controlling RA-inflammation-induced NETosis. High levels of ACPA and RF inducing NETosis can be detected in the serum of RA patients (43) and promote the further production of autoantigens in the form of citrullinated protein, resulting in persistent inflammation and tissue damage; abnormal NETs may exacerbate the expression of citrullinated autoantigens and immune-stimulating molecules, thereby promoting the development of epitopes associated with RA pathogenesis. In addition, the formation of NETs promotes the recruitment of various inflammatory factors to the joint inflammation site, increasing cartilage injury and promoting the development of RA. NETs can increase the production of related pro-inflammatory factors such as IL-1β and IL-18, further stimulating the formation of NETosis. These mechanisms contribute to inflammatory arthritis and joint destruction, thereby driving the onset of disease and the presentation of RA (44). Of course, the proper generation of NETs can activate the immunomodulatory response and alleviate the pathological process of RA by regulating T cells and dendritic cells, and NETs can also clear the apoptotic cells in joints and reduce the inflammatory response caused by these cells. We should strive to explore a balance point between optimal treatment and minimal impact and better clarify the relationship between RA and NETs. In the following article, we mainly explore the mechanism of the pathogenic effect of NETs on RA.

### 3.1 NETs induce the citrullination of RA

#### 3.1.1 NETs promote the ACPA citrullination of RA

Currently, definitive diagnosis of RA depends on the detection of RF and ACPA (43), among which ACPA is recognized as one of the most important diagnostic biomarkers with high specificity and sensitivity. The specificity of ACPA is crucial for immunopathology caused by autoantibodies. Studies have shown that ACPA isolated from RA patients not only reacts with broad-spectrum citrulline peptides with different affinities but also shows heterogeneity (45). There are multiple types of structural interactions between ACPA and its citrulline antigen. ACPA interacts with citrulline and amino acid side chains and is specifically recognized or “cross-reacted” to citrulline labeled on joint proteins, showing an arthritic effect (46). Ge et al. have demonstrated that ACPA can promote proteoglycan consumption of cartilage and aggravate joint inflammation through the cross-reaction of specific ACPA with articular cartilage (47).

Citrullinated antigens on NETs play a key role in the initiation and development of autoimmunity and ACPA (48). Citrulline is derived from arginine through a post-translational modification of peptidyl arginine deiminase (PAD) and thus converted to citrulline protein (49). Exposure of citrulline protein to NETs is a key driver of autoimmunity, leading to the formation of ACPA (50). Citrulline vimentin itself is present in stimulated neutrophils or NETs, and anti-citrulline vimentin autoantibodies can induce the formation of NETs (43). At the same time, the release of NETs can further aggravate the production of ACPA, resulting in the production of inflammatory molecules such as IL-6, IL-8, chemokines, and adhesion proteins. As a result, ACPA continuously promotes neutrophils to release arginine deaminase (PADI), which can in turn modify arginine to citcitline. Thus, a vicious cycle of autoantibodies is formed (51). Moreover, it is pointed out that the exposure of citrullinated antigens by NETs to the immune system with the same citrullinated antigens and inflammatory cytokines will perpetuate NETosis (52). In addition, RA synovial cells exhibit an unusual pattern of citrullination known as cellular “hypercitrullination”. Hypercitrullination is induced by perforin and membrane attack complex (MAC)-mediated membrane lysis pathways, which are activated in RA joints and produce ACPA (18, 53). It has also been shown that therapeutic anti-citrullinated protein autoantibody (tACPA, a therapeutic antibody that specifically recognizes citH2A and citH4) inhibits the formation of NETs in humans and mice induced by different physiological stimuli *in vivo* and *in vitro*, and that tACPA treatment prevents the release of NETs in a mouse model of CIA (54). In addition, NETs-derived NE can destroy cartilage structure and promote its citrullination, thereby increasing its immunogenicity and the production of autoantibodies and eventually leading to joint inflammation (55). Thus, NETs induce citrullination in RA patients and exacerbate RA symptoms.

#### 3.1.2 NETs promote histone citrullination

**Figure 2:**
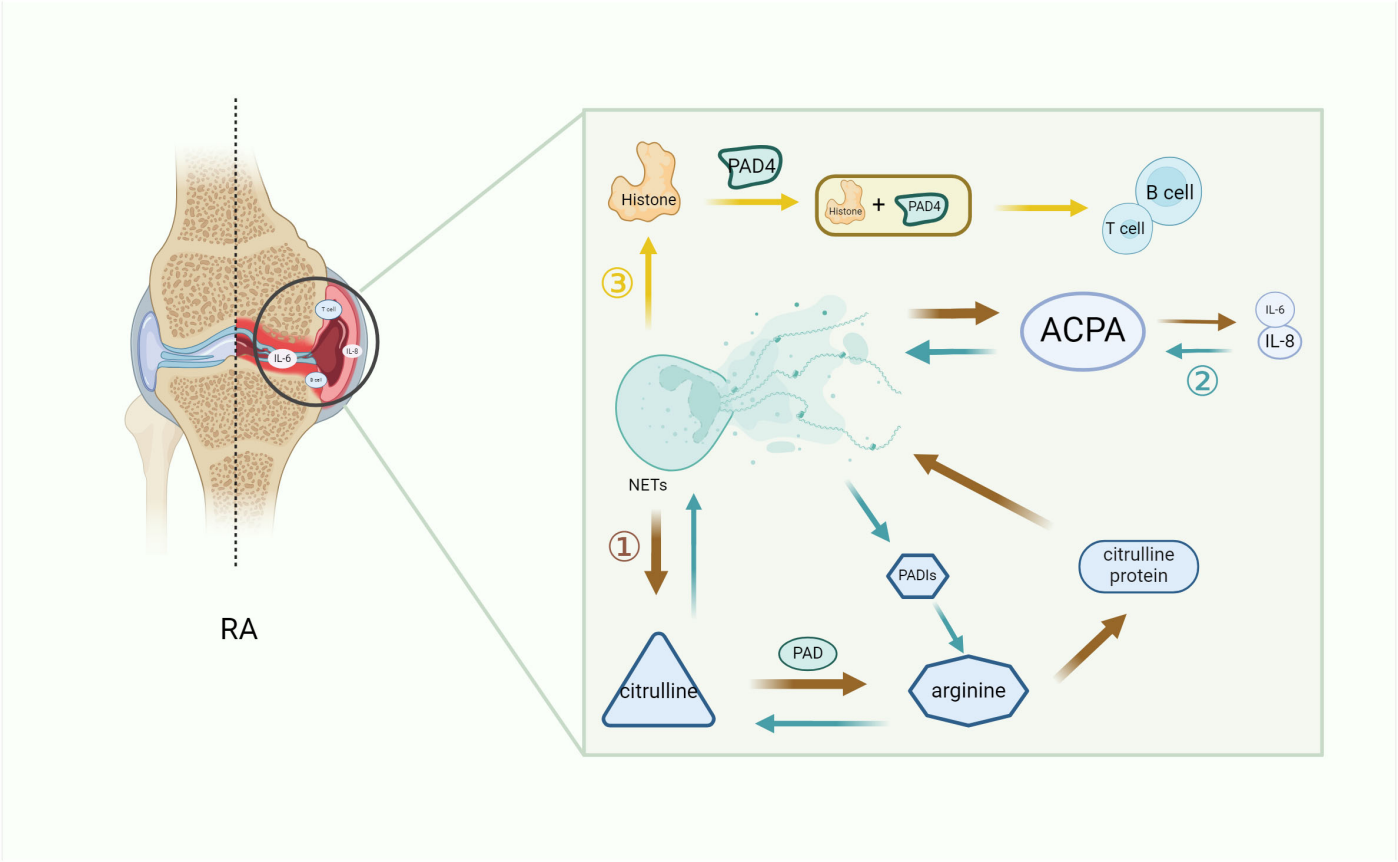
The key role of NETs in RA inflammation. NETs increase the secretion of immune cells and pro-inflammatory factors by promoting the citrullination of RA autoantibodies (1) and histones (3), while ACPA continuously promotes the formation of pro-inflammatory factors to promote citrullination (2), thus forming a vicious cycle and exacerbating the symptoms of RA. (1) Citrulline antigen in NETs is modified by PAD to form arginine, which promotes the production of citrulline protein and development of ACPA in NETs, thereby secreting pro-inflammatory factors and worsening RA. (2) The increase of pro-inflammatory factors leads to an increase in ACPA, and the reverse conversion of arginine into citrulline through PADIs leads to a vicious cycle of autoantibody formation. (3) The histones produced by NETs, which are citrullinated by PDA4, produce immune cells and aggravate RA. NETs, neutrophil extracellular trap; PADIs, arginine deaminase; IL-6, interleukin 6; IL-8, interleukin 8; B cell, B lymphocyte; T cell, T lymphocyte; PAD, peptidyl arginine deiminase; PAD4, peptidyl arginine deiminase 4; RA, rheumatoid arthritis.

Histone is an important component of NETs, and the histone levels in the synovial fluid of RA patients are increased compared with osteoarthritis patients. In neutrophils, PAD4-induced histone amination is a key step in NETosis, enabling the apparent release of active PDA4 into the synovial fluid of RA patients, promoting the production of autoantibodies and leading to inflammation (56). Additionally, extracellular histone plays a similar role. Owing to the inflammatory properties of NETosis and the characteristics of extracellular histone, the extracellular histone in RA synovial fluid interacts with other cells in the joint, amplifying the pro-inflammatory role of histone in RA inflammation (57). NETs that spontaneously express citrulline histone H3 (Cit-H3) have higher levels in the neutrophils of RA patients, which intensify the local production of ACPA and accelerate the development of inflammation (58, 59). In addition, NETs directly induce STAT3 phosphorylation by interacting with TLR2 expressed on T cells through histone, which promotes the differentiation and activity of TH17 cells in the presence of Th17-cell-promoting cytokines (60), while it is known that TH17 cells can promote the of recruitment neutrophils through IL-17. Thus, histone exacerbates inflammation in RA patients by increasing the recruitment of inflammatory cells to the lesion site (**Figure 2**).

### 3.2 NETs stimulate inflammatory factors to induce synovial inflammation in RA

In RA synovium, the activation of macrophage-like synoviocytes (MLSs) underlies an important source of cytokines and proteases (61). MLSs produce several pro-inflammatory factors, including IL-6, IL-8, and tumor necrosis factor (TNF)-α, while the involvement of TNF-α and IL-6 is the core of the pathogenesis of RA. Through a series of studies in TNF-knockout mice, Kruglov et al. revealed that membrane TNF (memTNF) possesses anti-arthritic protective function and inhibits self-reactive T cells (62). TNF can also regulate the progression of arthritis by promoting the activation of Fibroblast-like synoviocytes (FLSs) and inducing autoantibodies. On the other hand, T-cell-derived TNF has been shown to play a defensive role by mobilizing the development of auto-reactive T cells (63). NETs can also directly mobilize T-cell function, induce an increase in CD69 and CD25 expression, and lead to the activation of CD4+T cells, which have the potential to form inflammation and adaptive immune response (64). Studies have shown that in CD4+T lymphocytes of RA patients, CD28-CD4+T cells overexpress BcI-2, leading to atypical clonal expansion of autoimmune T cells (65). A large number of T cells infiltrate the synovial membrane and interact with dendritic cells (DC), monocytes and macrophages to jointly mediate synovial inflammation. At the same time, the localization of NETs by synovial macrophages is inseparable from the signaling of B cells. Studies have shown that NE contained in NETs promotes the secretion of inflammatory cytokines in macrophages by activating the nuclear factor K light chain enhancer (NF-kB) signaling pathway of B cells, and induces macrophage inflammation to aggravate RA synovial inflammation in the same way as Rab5a (66). These studies all indicate the important role of TNF-α, immune cells, and related inflammatory factors in the progression of RA. The production of NETs can promote the secretion of IL-6 (67) and TNF-α in macrophages, thus aggravating RA synovitis. At the same time, chemokines IL-8 and IL-17 contribute to the stimulation and aggregation of neutrophils to inflammatory sites. Brinkmann et al. found that IL-8 can stimulate neutrophils to produce NETs (8, 18). Ritika Khandpur et al. compared RA neutrophils with control neutrophils and found that RA neutrophils are more likely to develop NETosis after exposure to IL-17A and TNF-α (43). Interestingly, NETs have been observed to be internalized by macrophages and promote the secretion of cytokines, and the inhibition of NETs can alleviate inflammation by reducing the secretion of cytokines from macrophages (66). The production of NETs promotes the secretion of pro-inflammatory factors in MLSs, while exacerbating the distribution of inherently existing inflammatory factors in the diseased joints and exacerbating synovial inflammation.

### 3.3 NETs aggravate articular cartilage injury in RA

RA is characterized by persistent synovial inflammation, leading to articular cartilage and bone injury. FLSs, as the main effectors in cartilage injury (68), exhibit an aggressive phenotype and produce pathogenic inflammatory mediators, such as cytokines. Additionally, they produce mechanodegrading enzymes, especially matrix metalloproteinases and histoproteinases (69), to promote local inflammation and disease persistence (70). FLSs exhibit key immunomodulatory effects through direct interactions between secreted inflammatory cytokines and direct synovial-infiltrating immune cells (71). Specifically, the lining layer of the synovium of the joint is hyperplasic in RA, sometimes to a depth of 10–15 cells, and at the joint boundary, the lining layer may become a “pannus” tissue rich in FLSs and osteoclasts. Pannus is composed of macrophages, FLSs, dendritic cells or plasma cells, and mast cells, which mediate injury and erosion in the later stages of disease (61). Moreover, it invades adjacent articular cartilage and subchondral bone to produce inflammatory cytokines and chemokines, resulting in clinical symptoms such as swelling and pain (72).

**Figure 3:**
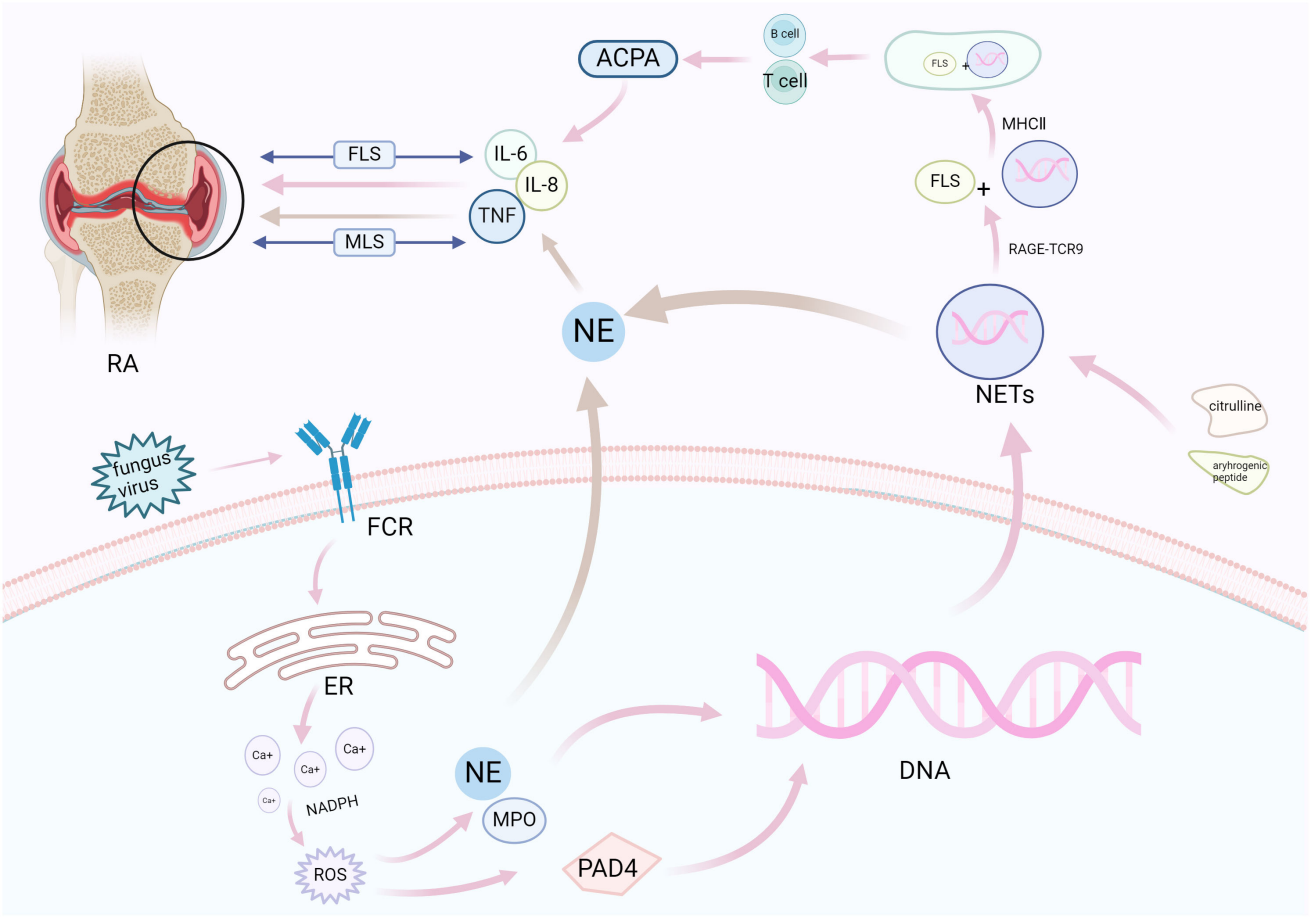
NETosis promotes synovial inflammation and joint injury in RA. Neutrophils respond to various stimuli to develop NETosis, which begins with the NADPH oxidase complex and promotes ROS production. ROS activates the hydrolytic activity of PAD4 and neutrophil elastase, causing excessive citrullination of PAD4 to promote nuclear DNA fragmentation, thereby releasing NETs. NETs bind to FLSs through related pathways under the action of citrulline and arthrogenic peptides (pink), release pro-inflammatory factors under the combined action of neutrophil elastase (brown), and aggravate synovial and cartilage injuries under the bidirectional action of FLS and MLS. FCR, fragment crystallizable receptor; ER, endoplasmic reticulum; NADPH, nicotinamide adenine dinucleotide phosphate oxidase; ROS, reactive oxygen species; PAD4, peptidylarginine deiminase 4; NE, neutrophil elastase; MPO, myeloperoxidase; NETs, neutrophil extracellular traps; NADPH, nicotinamide adenine dinucleotide phosphate; IL, interleukin; MHCII, MHC class II; ACPA, anti-citrullinated protein antibodies; FLSs, fibroblast-like synoviocytes; MLS, macrophage-like synovial cells; RA, rheumatoid arthritis.

Owing to the abundant infiltration of neutrophils in RA synovium, NETs are prone to form. The circulating neutrophils in RA patients are more likely to be stimulated by NETosis than those in healthy subjects, resulting in excessive innate and adaptive immune responses (73). NETs containing citrulline and arthrogenic peptide are internalized by FLSs via the RAGE-TLR9 endocytosis pathway, resulting in a pro-inflammatory phenotype in these cells. Once internalized, NETs promote the upregulation of MHC class II (MHCII) and load arthritogenic NET peptides into MHCII, which NETs then transport to FLS membranes where they are presented to antigen-specific T cells. This process promotes T-cell activation and B-cell response, leading to the production of ACPA and spread of inflammatory responses, eventually insulting cartilage injury (68). In short, the secretion of pro-inflammatory cytokines by FLSs exposed to extracellular traps may further increase NETosis, amplify exposure to citrulline autoantibodies, and promote the production of autoantibodies in patients, thus exacerbating cartilage injury (43). Carmona-Rivera et al. pre-incubated RA-FLSs with TLR antagonists, followed by exposure to RA-NETs, and found that the ability to internalize MPO (a molecule present in NETs) was impaired, demonstrating that FLSs internalize NETs via the RAGE-TLR9 axis and the induced pro-inflammatory features of FLSs are dependent on NETs internalization (68). Elastase in NETs can disrupt the cartilage matrix (50), and MMP8 and MMP9 have been found in RA-NETs to cause degradation of the cartilage matrix (74, 75), exacerbating cartilage injury. In addition, the immune complex formed by autoantibodies can activate macrophages to release pro-inflammatory factors, which amplify cartilage injury by releasing elastase from neutrophils (76) and trigger the occurrence of RA. NETs promote the rapid formation of osteoclasts by monocytes via the transduction of TLR4 and the NET-related protein pathway, directly affecting RA-related bone erosion (77). NETs stimulate the release of Rankl, inhibit the secretion of osteophosphorin in osteoblasts, and facilitate the formation of osteoclasts. Inhibition of NETs is a promising strategy for reducing bone erosion in patients with RA (78) (**Table 2**; **Figure 3**).

**Table 2:** Studies on the role of NETs in RA.

The role of NETs in RA		Main findings
Citrullination of RA was induced	Citrullination of autoantibody (ACPA)	Increased NETs was observed in the peripheral blood and synovium of patients with RA, as were externalizing citrulline autoantibodies, which play a pathogenic role in RA (43) In CIA mouse models, the release of NETs was inhibited after tACPA treatment (54)
	Histone citrullination	Citrullination of histones via PAD4 promotes the production of ACPA and NETs (59) In RA risk subjects compared with controls, the results showed that NETs of citrulline protein increased, promoting ACPA production and supporting the underlying mechanism of ACPA production (58)
Stimulating inflammatory factors induced synovial inflammation of RA		IL-6 and other inflammatory factors are key factors in the pathogenesis of RA (62) Neutrophils are stimulated by IL-8 to promote the production of NETs (8) NETs are internalized by macrophages, which further produce inflammatory factors (66)
Aggravate synovial inflammation of RA		FLSs internalize NETs containing arthrogenic peptides via the RAGE-TLR9 axis, promoting inflammatory response and cartilage damage (68) MMP plays an important role in cartilage degradation. There is a need to investigate the development of Antibody (Ab)-induced arthritis in MMP-2/MMP-9 mice (75) Elastase derived from NETs was found in the peripheral blood of RA patients to magnify cartilage injury and aggravate RA inflammation (76)

## 4 NETs may be the key target for controlling RA

The relevant mechanisms proposed in this study indicate that inhibition of NETs can be a key entry point for the treatment of RA. Quite a few reports have pointed out that the use of NET inhibitors can markedly mitigate joint swelling and inflammation. Alleviating the pathological process of RA by inhibiting the generation of NETs is also a hot topic at present. However, owing to the diversity and uncertainty of the pathways generating NETs in RA, there is still much room for further research on NETs. The increased level of NETs in synovial fluid may be attributed to the impaired or inhibited activity of DNA-1, and DNase can effectively eliminate NETs and inhibit the proteolytic enzyme activity of NE (79). Hence, DNase I is considered for the treatment of RA (80). In addition, the use of PAD4 inhibitors in K/B×N mouse models can effectively inhibit the formation of NETs, alleviate the degree of joint swelling, and significantly reduce the fibrous structure of NETs (81). As NETs are involved in the pathological process of RA, scholars have described a monoclonal antibody (Cit-013) with a high affinity for Cit-H2 and H4, which bears significant anti-inflammatory effects and inhibits the metabolism of NETs *in vivo*. Epitope detection of RA synovium has proven the potential of Cit-013 in targeting excess citrullination in RA and provided new insights into the development of antagonists for NETs in the treatment of RA (82).

In addition, a large number of studies have demonstrated the feasibility of natural extracts targeting NETs to improve RA. Quercetin inhibits neutrophil infiltration and diminishes plasma inflammatory cytokines, promotes apoptosis of activated neutrophils, and inhibits NETosis by regulating autophagy in RA mice, making it an ideal candidate in the management of RA (83). Emodin, a natural anthraquinone derivative, accelerates apoptosis but inhibits autophagy and NET formation by reducing IL-6 and TNF-α in mice with adjuvant arthritis (AA) (84). In CIA mice, polydatin (PD) reduces NET formation in myeloid neutrophils and RA patients, and treatment with PD reduces NET deposition in the ankle (85). In addition, apocynin and paeonol (APPA) can downregulate ROS levels, reduce NET formation, and inhibit TNF-α-induced cell conduction, fulfilling an anti-inflammatory role and exerting a potential therapeutic effect in RA (86). Tanshinone IIA (TIIA), by virtue of its favorable antioxidant and anti-inflammatory effects, can effectively diminish IL-6 and TNF-α levels in a mouse model of AA, reduce the formation and infiltration of NETs, and alleviate cartilage erosion of mouse ankle joint (87). *Cayratia albifolia* C.L.Li (CAC), as a widely used herbal medicine in autoimmune diseases, can increase anti-inflammatory activity by regulating the PI3K-Akt-mTOR signaling pathway to target NETs and macrophages, which effectively alleviates inflammatory damage in the hind paws of CIA rats and represents another effective candidate for the treatment of RA (88).

In addition, calprotectin released by NETs is identified as an inflammatory marker of RA. Calprotectin is an antimicrobial peptide mainly secreted by neutrophils, which can regulate intracellular calcium ion concentration and play an important role in immune response (89). The activation of neutrophils elevates the intracellular calcium concentration. Neutrophils release S100A8/A9 through NETosis, and the high level of S100A8/A9 found in the serum of RA patients may be caused by the disorder of NETosis (90). S100A8/A9 has been recognized as another important inflammatory marker of RA (91, 92), highlighting the exact contribution of NETosis. Calprotectin has been previously reported to increase inflammatory responses by inducing peripheral blood mononuclear cells to secrete cytokines and activating β2 integrins of neutrophils (93). Therefore, in the diagnosis of RA, calcarein protein can be tried as a related inflammatory marker, and the development of NETs as potential biomarkers for the diagnosis and disease monitoring of RA is still an area that needs our continuous efforts. Although preclinical studies have shown that the effective inhibition of NETs is an important measure in the treatment of RA, most relevant studies remain in preclinical studies, and approved drugs targeting NETs are very scarce, such as drugs targeting PAD4 that have not been approved for human use (94). We should strive to find drugs that can specifically block NETs and explore different pathways to inhibit NETs more deeply to better promote the development of clinical therapies for RA.

## 5 Discussion

As the product of neutrophils, NETs exert anti-inflammatory effects and resist inflammation by acting as microorganisms that capture and kill pathogens. On the other hand, the production of NETs under improper activation leads to tissue damage and recruits relevant pro-inflammatory factors or proteins to promote the release of inflammation, thereby amplifying joint and synovial inflammation and insulting cartilage injury in RA. Under this mechanism, the targeting of NETs has been demonstrated as an important therapeutic strategy in the management of RA. Repressing the improper activation of NETs induced by neutrophils can alleviate joint inflammation in RA. One of the anti-inflammatory mechanisms of methotrexate (MTX), a common drug used in the treatment of RA, is to reduce the production of cytokines by upregulating the level of adenosine on neutrophils, especially the inhibition of TNF-α and IL-1β, which reduces the accumulation of white blood cells in the inflammatory site (51). Infliximab can also reduce the activation of white blood cells in the synovium of RA patients to reduce the inflammatory response (6). It is also possible to develop anti-inflammatory targets for RA based on the key factors triggering NETs. For example, the generation of ROS is an indispensable link in NETosis. Although the generation mechanism of NETs in RA has not been fully elucidated, the NOX-dependent pathway is considered the key pathway (43). By reducing the activation of NOX and maintaining the stability of mitochondrial membrane, the generation of ROS can be reduced to block lipid peroxidation, further repressing the formation of NETs and release of inflammatory factors, thus slowing down the occurrence of RA inflammation. Moreover, NE also promotes the formation of NETs. Inhibition of NE can reduce NET-mediated tissue damage and improve the efficacy of drugs to prevent the formation of NETs, without the shock caused by endotoxin (95). Although the specific mechanism and method mentioned above still need to be further explored, they can provide new ideas that may contribute to solving the global issue of RA.

From another perspective, we can also start from the pathogeneses of RA, the most striking of which is the pro-inflammatory effect generated by the interaction of cytokines induced in several ways. In addition to the induction of cytokines by NETs, the activation of other cells can also promote the release of inflammatory factors and aggravate joint inflammation. For example, macrophages can be activated by a variety of pathways, including TLR ligands, soluble proteases, cholesterol derivatives, and immune complexes. ACPA and RF act through FcgR1 and FcgR3 expressed on synovial macrophages, inducing high levels of cytokines to produce pro-inflammatory effects (96). Whether NETs co-activate inflammatory factors with other cells and their association with other cells need to be explored continuously. Further studies on the mechanism of RA cytokines can also provide a deeper understanding of the pathogenesis of RA and contribute to the development of new therapeutic targets.

Currently, cell therapy based on mesenchymal stem cells (MSCs) has become an effective treatment for autoimmune diseases, which increases the severity of arthritis in RA mouse models by inhibiting inflammatory factors and promoting the differentiation of regulatory T cells (TREGs) (97). Human gingival-derived mesenchymal stem cells (GMSCs) can inhibit the formation of NETs by inhibiting the PGE2-KA-ERK signaling pathway to improve RA (98). This method provides a new direction for NET targeting research in RA and is worthy of further study.

However, at present, the treatment of RA with NETs as the target is mostly at the research stage of animal models, e.g., PAD4 inhibitors. Although such drugs have been applied in animal and cell studies, there are still many shortcomings, and further studies are needed in the aspects of body selectivity and adverse reactions. Moreover, there is a large amount of evidence of citrullination in human RA, but in the serum transfer model, mice lacking PAD4 still show joint inflammation, indicating that citrullination of the NET component is not required in this model, and there is a great difference in the induction of NETs between mice and humans (99). Therefore, how to reduce the difference between animal models and human RA? And whether the targeted therapy of NETs in animal models can be realized in humans is still a question that we need to explore.

In this study, we mainly introduced the pathogenic role of NETs in RA, and a number of studies have shown that inhibiting the production of NETs has a good effect on alleviating the inflammatory symptoms of RA. However, some studies have shown that the use of PAD4 inhibitors in the Neonates with necrotizing enterocolitis (NEC) model characterized by bacteremia reduces the production of NETs but aggravates the inflammatory response of mice. At the same time, bacterial translocation and mortality were increased (100), and related studies found that PAD4-dependent NETs were essential for the intestinal clearance of *Citrobacter rodentum* intestinal infection, suggesting the beneficial effect of NETs (101). These conflicting results suggest that the effect of NETs on different diseases is not static, and future studies are needed to further investigate the role of NETs in RA and other diseases and explore the beneficial therapeutic effects of NETs in various diseases, including RA.

RA is a global concern, and its exact pathogenesis needs further exploration. This article briefly summarizes the production of NETs and their influence on the pathogenesis of RA, and proposes the key role of NETs in the pathogenesis of RA. To sum up, given the critical implication of NETs in RA, the targeting of NETs is a promising application in the treatment of RA. In-depth exploration of the pathogenesis of NETs in RA is expected to lead to the development of more effective treatment strategies for RA.
